# Drosophila Lipase 3 Mediates the Metabolic Response to Starvation and Aging

**DOI:** 10.3389/fragi.2022.800153

**Published:** 2022-02-14

**Authors:** Lea Hänschke, Christoph Heier, Santiago José Maya Palacios, Huseyin Erdem Özek, Christoph Thiele, Reinhard Bauer, Ronald P. Kühnlein, Margret H. Bülow

**Affiliations:** ^1^ Life and Medical Sciences (LIMES) Institute, University of Bonn, Bonn, Germany; ^2^ Institute of Molecular Biosciences, University of Graz, Graz, Austria; ^3^ BioTechMed- Graz, Graz, Austria; ^4^ Field of Excellence BioHealth, University of Graz, Graz, Austria

**Keywords:** *Drosophila*, lipid, phosphatidylinositol, lifespan, lipidomics, metabolism, *lipase*

## Abstract

The human *LIPA* gene encodes for the enzyme lysosomal acid lipase, which hydrolyzes cholesteryl ester and triacylglycerol. Lysosomal acid lipase deficiency results in Wolman disease and cholesteryl ester storage disease. The *Drosophila* genome encodes for two LIPA orthologs, Magro and Lipase 3. Magro is a gut lipase that hydrolyzes triacylglycerides, while Lipase 3 lacks characterization based on mutant phenotypes. We found previously that *Lipase 3* transcription is highly induced in mutants with defects in peroxisome biogenesis, but the conditions that allow a similar induction in wildtypic flies are not known. Here we show that *Lipase 3* is drastically upregulated in starved larvae and starved female flies, as well as in aged male flies. We generated a lipase 3 mutant that shows sex-specific starvation resistance and a trend to lifespan extension. Using lipidomics, we demonstrate that Lipase 3 mutants accumulate phosphatidylinositol, but neither triacylglycerol nor diacylglycerol. Our study suggests that, in contrast to its mammalian homolog LIPA, Lipase 3 is a putative phospholipase that is upregulated under extreme conditions like prolonged nutrient deprivation and aging.

## Introduction

Caloric restriction and intermittent fasting induce transcriptional programs that lead to break-down of storage fat and glycogen and induce autophagy. The latter functions as a self-renewal program to the cell and is accepted as one of the drivers of lifespan extension and the health-promoting effects of dietary restriction (Madeo et al., 2015). By contrast, the constant undersupply with important macro- and micronutrients, as well as long periods of starvation, are indubitably detrimental (Martins et al., 2011). Lipases are important effectors of the response to starvation by mobilizing lipid stores such as triacylglycerides. Across species, lipid mobilization takes place already after short periods of nutrient restriction to maintain the supply of the brain when glucose is limiting (Izumida et al., 2013). The activity of lipases that are involved in the breakdown of storage fat decreases with age, and their activation, e.g. by dietary restriction, is associated with lifespan extension. By contrast, lipases that regulate phospholipids in the brain increase in age-related neurodegenerative diseases (Oliveira and Di Paolo, 2010).

Lipases are essential for animal metabolism but sequence-based predictions of substrate specificity and organismal function of these enzymes are challenging. The human *lipase A (LIPA)* gene encodes the lysosomal acid lipase (LAL), which hydrolyses triacylglycerol (TAG) and cholesteryl ester (CE). Mutations in *LIPA* lead to lysosomal acid lipase deficiency, which can manifest as two clinical spectra: Wolman disease, which affects infants, and cholesteryl ester storage disease (CESD), which has a later onset. Both disease spectra are characterized by progressive accumulation of TAG and CE, leading to liver disease and early lethality (Witeck et al., 2021). Expression of LIPA under nutrient restriction is regulated by Forkhead box protein O1 (FOXO1), the main downstream effector of insulin signalling, and by transcription factor EB (TFEB), a regulator of autophagy by promoting lysosome biogenesis (Li and Zhang, 2019). LAL overexpression promotes hepatic inflammation under high fat diet and impairs lysosomal lipophagy (Lopresti et al., 2021), suggesting that LAL needs to be tightly regulated to ensure lipid homeostasis.

Research in the fruit fly *Drosophila melanogaster* has helped to elucidate the mechanisms of energy homeostasis and of metabolic diseases (Chatterjee and Perrimon, 2021). The genome of model organism *Drosophila melanogaster* encodes two lipases with homology to LIPA: Magro and lipase 3 (Lip3). Magro is not a lysosomal lipase but a gastric lipase that is required for the digestion of TAG and CE (Sieber and Thummel, 2012). It is regulated by Foxo and its expression is strongly reduced in old flies, leading to disturbed gut lipid homeostasis during aging (Karpac et al., 2013). Lip3 was initially identified as an acid lipase gene predominantly expressed during larval stages (Pistillo et al., 1998). Subsequently, the gene was characterized as transcriptional starvation marker. In larvae, *Lip3* expression is upregulated after 4 h starvation, but remains repressed under amino acid deprivation in the presence of carbohydrates (Zinke et al., 2002). *Lip3* is a target gene of Hepatocyte nuclear factor 4 (Hnf4), a transcription factor that regulates lipid mobilization and mitochondrial *ß*-oxidation upon starvation (Palanker et al., 2009), and its starvation-dependent induction on nuclear translocation of Lipin, a protein required for lipid homeostasis (Hood et al., 2020).

We described previously that *Lip3* is drastically induced in *Drosophila* mutants lacking the ceramide synthase homolog Schlank (Bauer et al., 2009). Next to the Schlank enzyme function in ceramide synthesis, the protein contains a DNA-binding domain and acts as transcriptional repressor. Under fed conditions, Schlank shuttles from the endoplasmic reticulum (ER) to the nuclear membrane and represses the transcription of *Lip3* and *magro*. Upon starvation Schlank leaves the nucleus and thereby releases its repression of *Lip3* (Sociale et al., 2018). *Lip3* is also drastically induced in mutants that lack peroxisomes. Peroxisomes are vital lipid-metabolizing organelles, and we previously showed that lipid imbalance caused by absence of peroxisomes (Pex19 mutants) induces the translocation of Schlank from the nuclear membrane to the ER, thereby releasing *Lip3* repression. Restoring lipid balance by dietary intervention, or by introduction of a Schlank variant that is constitutively nuclear, is sufficient to reduce *Lip3* transcript levels (Bülow et al., 2018; Sellin et al., 2018). High *Lip3* expression results in the accumulation of free fatty acids (FFA) in mitochondria of Pex19 mutants and contribute to their lethality (Bülow et al., 2018).

Dietary restriction reduces insulin signalling and promotes lifespan extension, but genetic conditions under which *Lip3* transcription is highly induced are pathologic. Here we investigate wild type flies under conditions that trigger extreme *Lip3* upregulation similar to Pex19 and Schlank mutants. Our data reveal substantial differences of the evolutionarily related *Drosophila* Lip3 and human LIPA lipase with respect to biochemical activity, regulation and biological function. We show that *Lip3* is induced in a sex-specific manner under prolonged starvation and aging. We hypothesize that Lip3 hydrolyzes phospholipids, specifically phosphatidylinositol (PI) rather than storage lipids TAG and CE. Lip3 mutants show extended lifespan and altered brain PI metabolism. Our study suggests that Lip3 is not a canonical Foxo-regulated lipase, but induced under extreme, and thus potentially pathological, conditions.

## Materials and Methods

### Fly Husbandry

Flies were reared on standard cornmeal food (130 g yarn agar, 248 g Baker’s yeast, 1223 g Cornmeal and 1.5 L sugar beet syrup in 20 L distilled water) and kept in a 25°C incubator with light-dark-cycle. Lip3 mutants were created by CRISPR/Cas9-mediated genome editing by non-homologous end-joining. Oligos used for Cas9 targeting of the *Drosophila* lip3 locus (CG8823) were sense 5′-CTT​CGA​CCA​TTT​TAC​TGG​TCG​GAT-3′ and antisense 5′-AAA​CAT​CCG​ACC​AGT​AAA​ATG​GTC-3’. Both oligos were annealed with a polynucleotide kinase to phosphorylate the oligo ends. Annealing was done at 37°C for 30 min, 95°c for 5 min, then temperature ramp to 25°C at a rate of 0.1°C per second. Annealed oligos were cloned into the pU6-BbsI-chiRNA vector *via* BbsI restriction sites and transformed into DH5α *E.coli.* Vector DNA was isolated from positive clones and verified *via* sequencing. Midi-prep purified vector DNA was sent to BestGene Inc., CA, USA for injection services in *Drosophila* embryos. Injections were done in wildtypic flies crossed with a Cas9-line carrying a CyO (curly of Oster) wing marker. For genotyping by high resolution melting analysis (HRMA), we used the primers 5′- ACT​GCG​ATG​ACA​AGA​GGA​GC-3′ and 5′- acG​CAA​TCA​GAA​ATA​GGC​CGA-3′. For keeping the newly generated fly line, flies were crossed to a balancer fly line for the third chromosome (TM3, Sb, Dfd GFP).

For lifespan analysis, isogenized flies were separated by sex and transferred to Longevity (LG) food (37.5 g Baker’s yeast, 10 g Agar Kobe I (Carl Roth, Cat# 5210.1), 300 ml dH2O; 3 ml of a 10% Nipagin (in 70% EtOH) and 37.5 g glucose were added after autoclaving) with 20 flies per tube. Number of viable and dead flies was controlled every day and LG food was changed every two days.

For starvation induction, larvae of the different genotypes (WT, Lip3 or Lip3-GFP-reporter line) were transferred to empty cell culture dishes with only PBS-soaked filter paper in it. Larvae were kept on these plates in the 25°C incubator for the indicated hours.

### Lipid Substrates

1,2-dioleoyl-*rac*-glycerol (Cat#: D8397), 1-oleoyl-*rac*-glycerol (Cat#: M7765), 1,2-dioleoyl-*sn*-glycero-3-phosphocholine (Cat#: P6354), soybean phosphatidylinositol (Cat#: 79401), and trimyristoylglycerol (Cat#: T5141) were from Sigma Aldrich.

### Molecular Cloning

A construct for the expression of LIPA-FLAG was created by amplifying the open reading frame of the human *LIPA* gene using the primers 5′-TAA​TGC​GGC​CGC​GCC​ACC​ATG​AAA​ATG​CGG​TTC​TTG-3′ and 5′-TAA​TGT​CGA​CCT​GAT​ATT​TCC​TCA​TTA​G-3′ and a commercially available plasmid (Origene #RC201637) as a template. The PCR product and a pCMV-FLAG 5.1 vector (Sigma Aldrich E6908) were digested with NotI and SalI, purified by agarose gel electrophoresis, and ligated. The open reading frame of the *Drosophila Lip3* gene was amplified using the primers 5′-CAG​AAT​TAA​GCT​TGC​GGC​CGC​GAA​TTC​GCC​ACC​ATG​ACA​AGA​GGA​GCG​TTA​AAA​GTG​AC-3′ and 5′-GTA​ATC​AGC​CCG​GGA​TCC​TCT​AGA​GTC​GAC​TTC​GTA​GGA​TTG​CAT​CTG​CTT​CAA​G-3′ and a pUAST-*Lip3* construct as template. The PCR product and a pCMV-FLAG 5.1 vector were digested with EcoRI and SalI, purified by agarose gel electrophoresis, and ligated. The open reading frame of *EGFP* was amplified using the primers 5′-GAT​CGA​ATT​CGC​CAC​CAT​GGT​GAG​CAA​G-3′ and 5′-GAT​CGG​ATC​CCT​TGT​ACA​GCT​CGT​CCA​T-3′ and a pEGFP-N1 vector as template. The PCR product and a pCMV-FLAG 5.1 vector were digested with EcoRI and BamHI, purified by agarose gel electrophoresis, and ligated.

### Cell Culture

Proteins were expressed in mammalian tissue culture cells and prepared for lipid hydrolase assays as described in Heier et al. (Heier et al., 2021). In brief, COS-7 cells were cultured in Dulbecco’s modified Eagle’s medium (Gibco Thermo Fisher Scientific) supplemented with 10% fetal bovine serum, 100 units/ml penicillin, and 100 μg/ml streptomycin at 37°C, 95% humidity, and 5% CO_2_. Cells were transfected with DNA complexed to Metafectene (Biontex GmbH) according to the manufacturer’s instructions and used for experiments 24 h thereafter. Cell culture samples for lipid hydrolase assays were prepared by sonication of COS-7 cells in 0.25 M sucrose, 1 mM EDTA, 1 mM DTT containing 20 μg/ml leupeptin, 2 μg/ml antipain, and 1 μg/ml pepstatin followed by centrifugation at 4°C and 1,000 × *g*. Protein concentrations of post-nuclear supernatants were determined using the Bradford protein assay with BSA as standard according to the manufacturer’s instructions (Bio-Rad).

### Lipid Hydrolase Assays

Monoacylglycerol (MAG) and diacylglycerol (DAG) hydrolase activities were assayed as described in Heier et al. (Heier et al., 2021) using either a Bis-trispropane buffer pH 7.5 or a sodium acetate buffer pH 5. Trimyristoylglycerol was emulsified by sonication at a concentration of 0.3 mM with 37 µM 1,2-dioleoyl-*sn*-glycero-3-phosphocholine and 11 µM phosphatidylinositol followed by the addition of essentially fatty acid free BSA to a final concentration of 2%. 1,2-dioleoyl-*sn*-glycero-3-phosphocholine was emulsified by sonication at a concentration of 0.3 mM with 4.8 mM CHAPS. The release of fatty acids from all substrates was quantified with a colorimetric kit (NEFA-HR(2) reagent, Cat#: 999–34691, Cat#: 995–34791, Cat#: 991–34891, Cat#: 993–35191, and Cat#: 276–76491, Fujifilm Wako Diagnostics, Mountain View, CA).

### Lipidomics

Lipidomics analysis of 5 individual 3rd instar larvae of each genotype was performed in a Thermo Scientific Q Exactive Plus Hybrid quadrupole-orbitrap mass spectrometer as described previously (Thiele et al., 2019). Reads were normalized to wet weight.

### Quantitative Real-Time PCR

Whole RNA of 5 individuals, or brains of 10 individuals, was isolated using Trizol reagent (Invitrogen, Cat.# 15596026). Tissue was homogenized using a Precellys 24 homogenizer (peqlab). Transcription to cDNA was performed using the Luna^®^ universal One-Step RT-qPCR Kit (New England Biolabs, Cat.#E3005). Quantitative PCR was performed with a CFX Connect cycler (biorad). A minus-RT was analyzed in a PCR for each cDNA. Quantitative PCR was performed with a CFX Connect cycler (biorad) using Luna^®^ universal qPCR Master Mix (New England Biolabs, Cat.#E3003). Values were normalized against two house-keeping genes (actin5c and rp49), and against matching control condition (e.g. female brain to female body, ΔΔCq). Each experiment was repeated at least 4 times. Primers were actin-5C 5′-GGC​CAT​CTC​CTG​CTC​AAA​GTC-3′ and 5′-GAT​CTG​GCT​GGT​CGC​GAT​T-3′, rp49 5′-TCC​TAC​CAG​CTT​CAA​GAT​GAC-3′ and 5′-CAC​GTT​GTG​CAC​CAG​GAA​CT-3′, lip3 5′-ATC​AAG​TCC​GCC​CAT​CTT​CT-3′ and 5′-CTC​TAT​GCC​CAA​ATC​CTG​CT-3′, Dgk (CG34361) 5′-ACT​CAC​ACT​CTT​CAA​GGA​CCT-3′ and 5′-CCC​AGG​GGA​ATC​ACT​CCG​A-3′, Cds (CG7962) 5′- ACA​AAA​CGC​CCG​AGA​TAT​TGG-3′ and 5′-CGA​AGC​CGC​AGA​TCA​TAA​TCC-3′, PIS (CG9245) 5′- GCC​GAG​CAC​GAT​AAC​GTC​TT-3′ and 5′- GGA​CAT​GAA​CCA​GAA​GGC​GA-3′.

### Imaging

For immunohistochemistry, we dissected guts from 2–5 day old female flies. Tissue was fixed for 30 min in 3.7% formaldehyde and washed with PBT before and after incubation with primary antibody and Alexa dye-coupled secondary antibody. The Lip3 antibody against the peptide AHLDFIWGTEARKYVYDEVLK was raised in rabbit (Davids Biotechnologie GmbH). Tissue was mounted in Fluoromont G and analyzed with a Zeiss LSM 710 confocal microscope using a 25× water objective (Plan-Neofluar, Zeiss). Image analysis was done in FIJI and corrected total cell fluorescence (CTCF) was determined with the following parameters: CTCF = Integrated Density—(region of interest X Mean fluorescence of background readings). For stainings of neutral lipids with Oil Red O, adult guts were dissected in PBS. Tissue was fixed for 20 min in 3.7% formaldehyde and washed with PBS. Before and after staining with a 0.1% Oil Red O solution in 60% isopropanol for 30 min, tissue was incubated for 5 min with 60% isopropanol. Tissue was washed with PBS, mounted in glycerol and immediately analyzed using an Olympus AX70 microscope. Molecular structures were drawn using BKChem software version 0.13.0 and further illustrated using Adobe Illustrator.

### Statistics

Bar charts represent mean and standard deviation. Boxes in box plots represent the interquartile range and median, whiskers represent minimum and maximum values. Green squares in box plots represent single data points. Red and blue squares in dot plots represent single data points from female (red) and male (blue) flies, respectively. We used Microsoft Excel for bar charts and dot plots and Origin Pro 8G for box plots. We used the software GraphPad Prism 9.0.1 for our statistical analyses and applied the following statistical tests: Two-sided Student’s t-test for normally distributed data in single comparisons, assuming heteroscedasticity. One-way ANOVA with Tukey-Kramer post-test was used for multiple comparisons. The lifespan curves were analyzed with Origin Pro 8G using the Mann-Whitney non-parametric test. The Kolmogorov-Smirnow test was applied to test normality, and Bartlett’s method was used to test for equal standard deviations within groups. 0.05 was used as alpha-value for the significance level. A minimum of 3 biological replicates was used for each analysis.

## Results

### Conditional *Lipase 3* Gene Expression Control by Lipid Metabolism Genes, Starvation, and Aging

**FIGURE 1:**
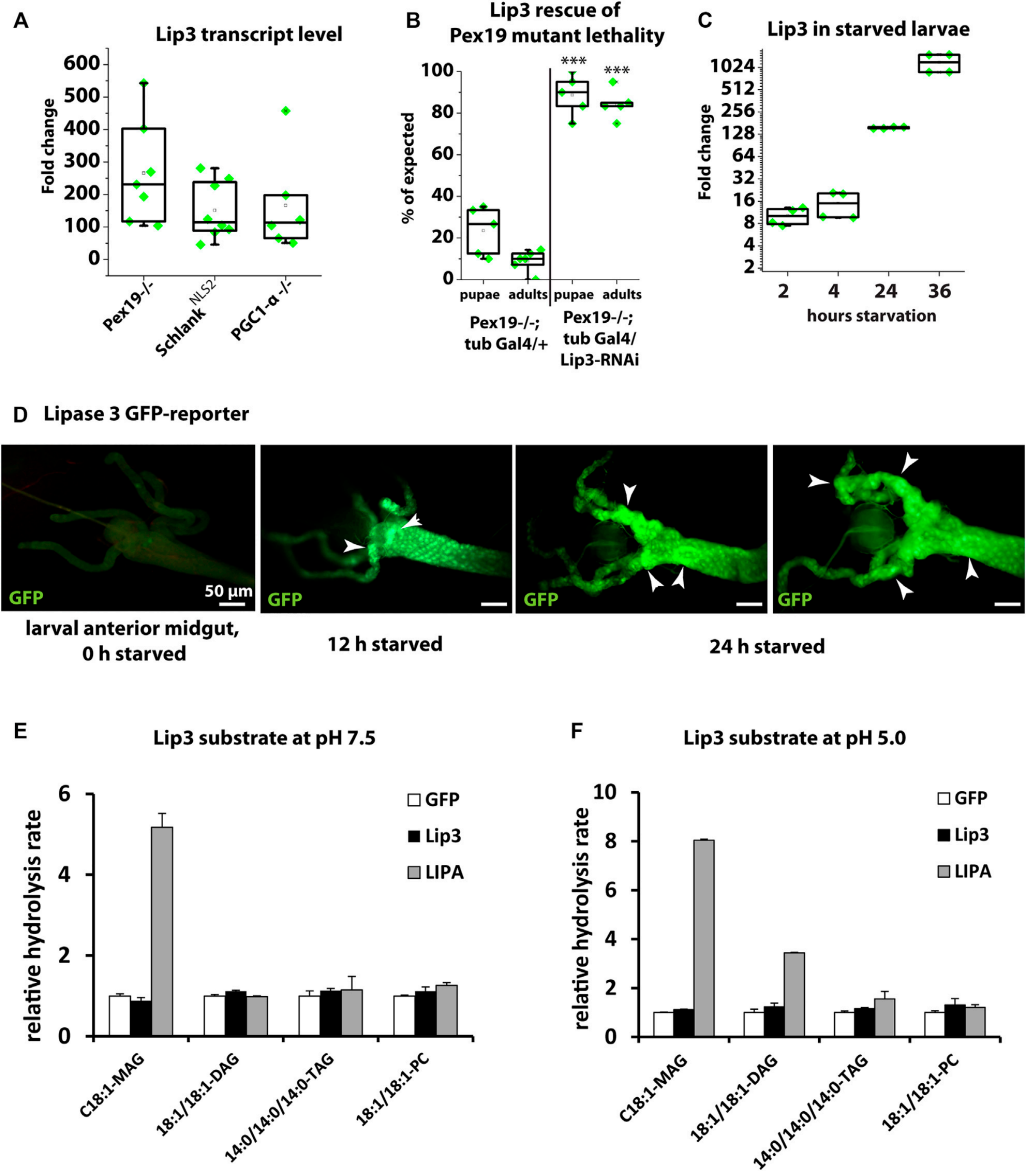
**(A)** Quantitative real-time PCR of Lip3 transcript of Pex19, Schlank-NLS2 and PGC1-α mutant larvae normalized to rp49 and actin-5c (ΔCq) housekeeping genes and wildtypic (w-) control larvae (ΔΔCq). **(B)** Rate of pupae and adults developing from 1st instar larvae. Genotypes are w*; pex19−/−; tubulin-Gal4/+ and w*; pex19−/−; tubulin-Gal4/UAS-Lip3-RNAi. **(C)** Quantitative real-time PCR of Lip3 expression in wildtypic starved larvae, normalized to rp49 and actin-5c housekeeping genes and relative to fed larvae. **(D)** Lip3 promoter-GFP reporter in anterior midguts of fed, 12 h starved, and 24 h starved early 3rd instar larvae. **(E, F)** Relative hydrolysis rate of Lip3 and LIPA (GFP as control) expressed in mammalian cell culture under neutral conditions (pH 7.5; E) and under acidic conditions (pH 5.0; F). Asterisks represent ***p < 0.001.

We described previously (Bülow et al., 2018; Sellin et al., 2018; Sociale et al., 2018) that Lip3 transcription is dramatically up-regulated in *Drosophila* mutants that affect lipid metabolism (Schlank) or organelle function (Pex19). In particular, *Lip3* transcription is upregulated by ∼250-fold in larvae mutant for the peroxisome biogenesis factor Peroxin 19 (Pex19, Figure 1A). The majority of Pex19 mutants die during larval development and compensatory downregulation of Lip3 by RNAi rescues their lethality (Figure 1B). Similarly, *Lip3* transcription is induced in Schlank^NLS2^ mutants (Figure 1A), which contain a functional ceramide synthase domain but lack the nuclear localization sequence NLS2, and therefore its transcriptional repressor activity (Sociale et al., 2018). Also mutants for the *Drosophila* homolog of Peroxisome proliferator-activated receptor-gamma coactivator (PGC)-1-alpha, called Spargel (Tiefenböck et al., 2010), upregulate Lip3 transcription to a similar extend (Figure 1A). This shows that genetic conditions that impair organelle function in the context of lipid homeostasis cause elevated Lip3 expression.

To understand whether Lip3 is also transcriptionally responsive to dietary impairment of lipid homeostasis, we starved early 3rd instar larvae for 2, 4, 24 and 36 h. While Lip3 transcription is only moderately induced after 2 and 4 h, the induction rises to ∼200 fold after 24 h, and to more than 1000 fold after 36 h (Figure 1C). To further analyze the spatial control of conditional Lip3 regulation, we monitored a transcriptional GFP reporter under control of the Lip3 promoter (Sociale et al., 2018) in the larval midgut, the main expression domain according to FlyAtlas (Chintapalli et al., 2007). While reporter expression was below the detection level in fed animals, a strong GFP signal was visible in the anterior midgut after 12 h of starvation, which accumulates and expands to the gastric cecae after 24 h of food deprivation (Figure 1D). This shows that high upregulation of Lip3 transcription is linked to prolonged rather than short periods of nutrient deprivation.

### Lipase 3 Substrate Specificity Differs From Human LIPA

Human lysosomal acid lipase hydrolyzes TAG and CE (Witeck et al., 2021). Bacterially expressed Lip3 has been shown to have weak TAG lipase activity on short-chain fatty acid glyceryl trioctanoate but no detectable activity on glyceryl trioleate, a more physiological substrate (Alfaro-Chávez et al., 2019). To further characterize the substrate specificity of *Drosophila* Lip3, we expressed LIPA, Lip3 and GFP as control in mammalian tissue culture cells. We determined the relative hydrolysis rate as the concentration of FFAs released from a given substrate under neutral (pH 7.5, Figure 1E) and acidic conditions (pH 5.0, Figure 1F, Heier et al., 2021). We found that LIPA uses C18:1 monoacylglycerol (MAG) and C18:1/C18:1 diacylglycerol (DAG) as substrate under acidic conditions, and also C14:0 TAG, albeit with lower activity. Consistent with its function as acid lipase, LIPA only hydrolyzed C18:1 MAG under neutral conditions. By contrast, Lip3 does not hydrolyze any of the tested substrates (Figures 1E,F), which suggests that Lip3 is not a functional homolog of LIPA, and raises the question what the Lip3 substrate is. To analyze this further, we generated a Lip3 mutant.

### Lipase 3 Mutant Larvae Accumulate Phosphatidylinositol

**FIGURE 2:**
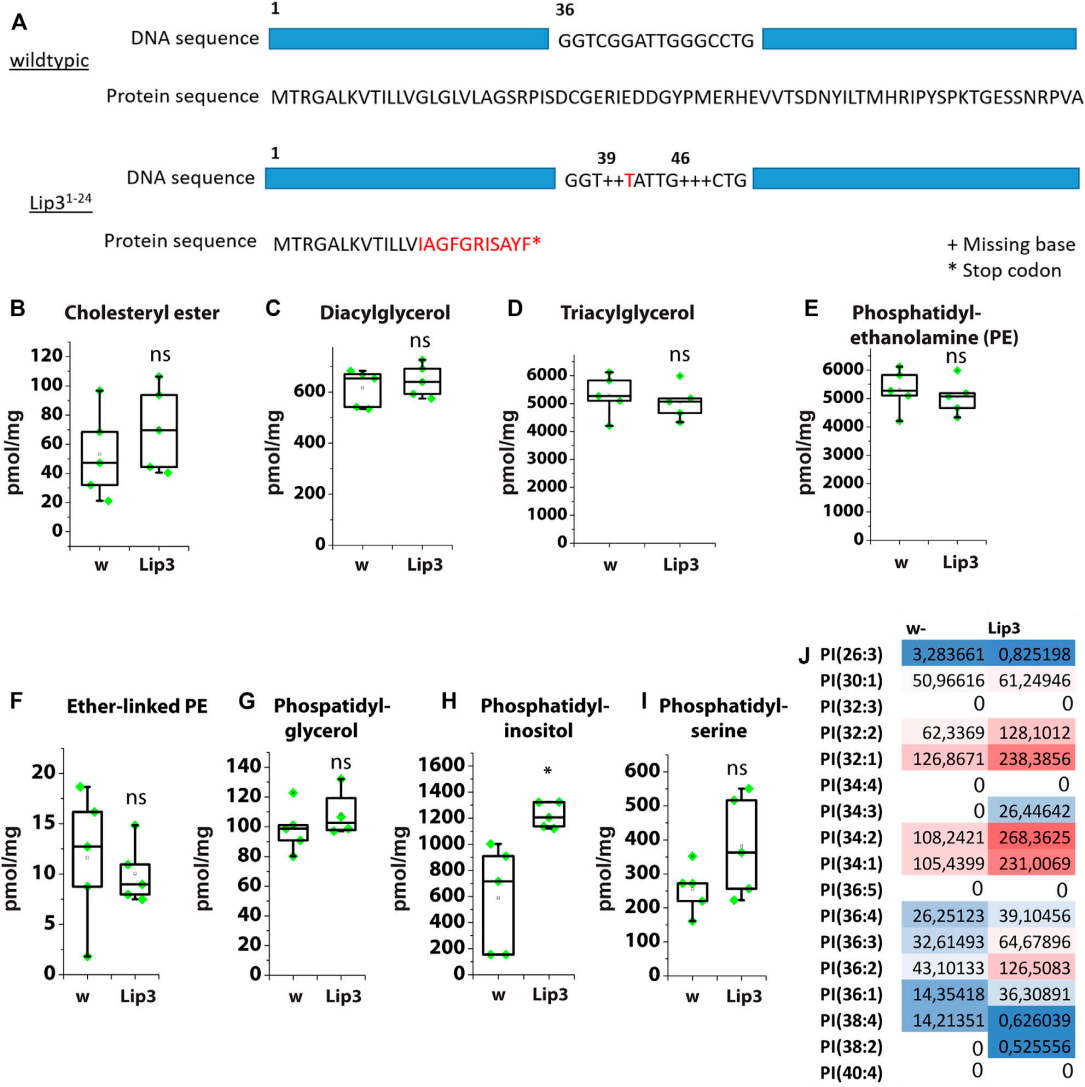
**(A)** Schematic showing the mutations of the Lip3 allele Lip3^1-24^. **(B)** Levels of cholesteryl ester (CE), **(C)** diacylglycerol (DAG), **(D)** triacylglycerol (TAG), **(E)** phosphatidylethanolamine (PE), **(F)** Ether-linked PE, **(G)** phosphatidylglycerol, **(H)** phosphatidylinositol, **(I)** phosphatidylserine as determined by lipidomics in control (w) and Lip3 mutant larvae. **(J)** PI species in w and Lip3 mutant larvae. Asterisks represent * *p* < 0.05, ** *p* < 0.01, *** *p* < 0.001, ns: not significant.

Ubiquitous overexpression of Lip3 induces FFA accumulation and reduces the survival rate of *Drosophila* larvae (Bülow et al., 2018). We generated a *Lip3* mutant by CRISPR/Cas9-mediated genome editing. The Lip3 mutant *Lip3* ^*1−24*^ has two deletions at bases 39 and 46, which result in a truncated Lip3 protein of 24 amino acids (Figure 2A), suggesting a functional null allele of the gene. *Lip3* ^*1−2*^4 mutants are viable and fertile.

We compared the lipidome of Lip3 mutant larvae to control larvae and found no significant difference in CE, DAG and TAG levels (Figures 2B–D). Similarly, the content of the phospholipids phosphatidylethanolamine (PE), ether-linked PE (PE-O), phosphatidylglycerol (PG) and phosphatidylserine (PS) is unchanged in Lip3 mutants, with the exception of phosphatidylinositol (PI) (Figures 2F–I). The levels of PI almost double from ∼700 pmol/mg in controls to ∼1200 pmol/mg in Lip3 mutants (Figure 2H). Detailed analysis of PI species shows that preferentially abundant PI with an average chain length between C16 and C18, such as PI(32:1), PI(34:1), PI(34:2) and PI(36:2) and low FA saturation are enriched, while the low abundant species such as PI(26:3) and PI(38:4) and higher FA saturation are reduced in Lip3 mutants (Figure 2J). It has been reported that in adult flies that were starved for 72 h, PI levels were reduced (Blumrich et al., 2021). Since we show that Lip3 is strongly induced upon prolonged starvation, increased Lip3 level might correlate with reduced PI levels under these conditions. Together, our results suggest that Lip3 controls PI metabolism either acting as phospholipase or *via* an indirect mechanism.

### Lipase 3 Is Highly Expressed in Starved and Old Adult Flies

**FIGURE 3:**
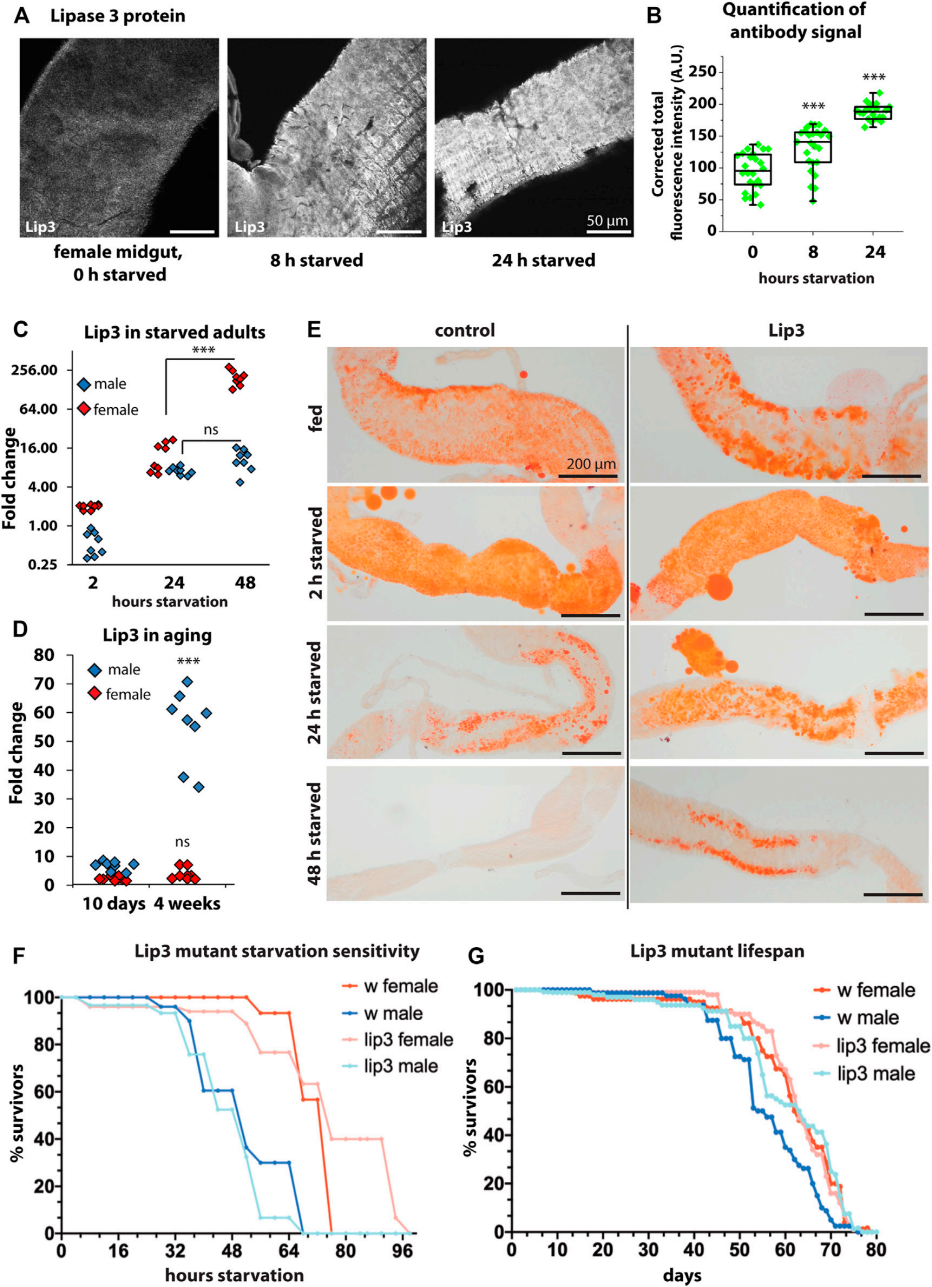
**(A)** Immunofluorescent staining of midguts with anti-Lip3 in fed, 8 h starved, and 24 h starved female flies. **(B)** Quantification of the fluorescent signals from A. **(C)** Quantitative real-time PCR of starved male and female flies, normalized to rp49 and actin-5c housekeeping genes and fed male and female flies, respectively. **(D)** Quantitative real-time PCR of 10 days (young) and 4 weeks old male and mated female flies, normalized to rp49 and actin-5c housekeeping genes and 2–5 day old male and female controls, respectively. **(E)** Oil RedO staining of midguts of female flies. **(F)** Starvation sensitivity assay, curves show average of three biological replicates of groups of 10 flies. Mann-Whitney non-parametric tests were applied for statistics but revealed no significant differences. **(G)** Longevity assay, curves show average of five biological replicates of groups of 20 flies. Mann-Whitney non-parametric test shows that the difference in lifespan between male wildtypes and Lip3 mutants is not quite significant (*p* = 0.056).

To analyze how the dynamic transcriptional pattern observed in larvae translates into protein expression, we raised an antibody against Lip3 (see Materials and Methods). Consistent with a high specificity for Lip3 the antibody signal is hardly detectable in the midgut of female Lip3 mutants compared to controls (Supplementary Figures S1B, C). In the midgut of fed female flies Lip3 is detectable at moderate levels. After 8 and 24 h of starvation Lip3 protein abundance progressively increases (Figures 3A,B, supplemental Figure S1A). We asked next if the Lip3 protein increase reflects starvation-induced transcription of Lip3 in adult flies as observed in larvae. While Lip3 transcripts hardly increase after 2 h of starvation compared to fed animals, gene expression rises to ∼8–16 fold in both male and female flies after 24 h of food deprivation. Notably, Lip3 transcription plateaus in male flies after 48 h of starvation, compared to fed males, but rises to ∼250 fold in females (compared to fed females). We conclude that prolonged starvation promotes high levels of Lip3 expression (Figure 3C, see Supplementary Figure S1D for Lip3 expression in Lip3 mutants), similar to genetic conditions that disturb lipid homeostasis (Figure 1A). Sex differences in Lip3 expression levels are not restricted to the starvation response but also detected in aging flies. Comparing young flies (10 days after hatching) to aged (4 weeks after hatching) flies revealed that Lip3 is exclusively upregulated in aged males but not in females (Figure 1D). Our results show that Lip3 is induced in a sex-specific manner under prolonged starvation and aging. Close correlation of the Lip3 transcriptional reporter and Lip3 proteins levels in the starvation response of the midgut predict a dynamic conditional regulation of the Lip3 enzymatic function.

The transcription factor Foxo is the major downstream effector of the insulin signaling pathway and shuttles from the cytoplasm to the nucleus already upon short starvation. Similarly, a transcription factor from the same protein family, Forkhead, shuttles from the cytoplasm to nucleus in response to Target of rapamycin (TOR) inhibition, i.e. amino acid starvation (Bülow et al., 2010; Bülow et al., 2015). To test if the induction of Lip3 under starvation depends on Forkhead box transcription factors, we ubiquitously expressed RNAi against Forkhead and a dominant-negative Foxo triple mutant (Foxo-TM) in adult females starved for 24 h. In both conditions Lip3 transcription was still induced upon starvation, suggesting that its regulation does not depend on Foxo and Forkhead (Supplementary Figure S1E). Reports on whether Lip3 transcription can be induced independently from Foxo are controversial: transheterozygous Foxo (foxo21/25) mutants were no longer able to induce Lip3 transcription upon starvation (Wang et al., 2011), while it was still induced in starved foxo21/w24 mutants (Becker et al., 2010). We suggest that Lip3 is not induced by canonical insulin/TOR signaling, but by signaling pathways that are only active upon more drastic conditions. We also suggest that the same signaling pathways are activated under peroxisome loss.

### Lipase 3 Mutants Show Sex-specific Starvation-Resistance and Lifespan Extension

We analyzed the starvation-induced mobilization of gut lipids in female flies by staining neutral lipids with Oil Red O (Figure 3E). Gut lipid levels appear similar in fed controls and *Lip3* mutants. After short starvation (2 h) lipid droplets are small and dispersed in the midgut of both genotypes. After 24 h of food deprivation midgut lipid stores are reduced in control females, but are still abundant in *Lip3* mutants. After 48 h of starvation, lipid stores are depleted in controls while they are reduced but still present in *Lip3* mutants. Currently it is unclear whether residual midgut lipid content after starvation in *Lip3* mutants compared to controls reflect pre-starvation differences in lipid storage unresolved by Oil Red O staining or whether these differences are the consequence of lipolysis impairment in the lipase mutant. Since both factors impact on starvation survival time in the TAG lipase Brummer (Grönke et al., 2005), we tested the starvation sensitivity of female *Lip3* mutants and found that, while the 50% survival rate is unchanged, the maximum survival time without nutrition increases (Figure 3F). This is consistent with our finding that *Lip3* is upregulated specifically in female flies under prolonged starvation. In contrast, male *Lip3* mutants are slightly more sensitive to starvation than control males consistent with the finding that male flies fail to mount a persistent transcriptional starvation response. Next, we asked if the loss of Lip3 function would affect the lifespan of flies. We found that *Lip3* mutant females have a similar lifespan compared to control females, while the lifespan of male *Lip3* mutant flies is extended, although not quite significantly (Figure 3G). This is consistent with our finding that *Lip3* is upregulated in aged male but not female flies.

### Brain Phosphatidylinositol Metabolism Is Altered in *Lipase 3* Mutants in a Sex-specific Manner

**FIGURE 4:**
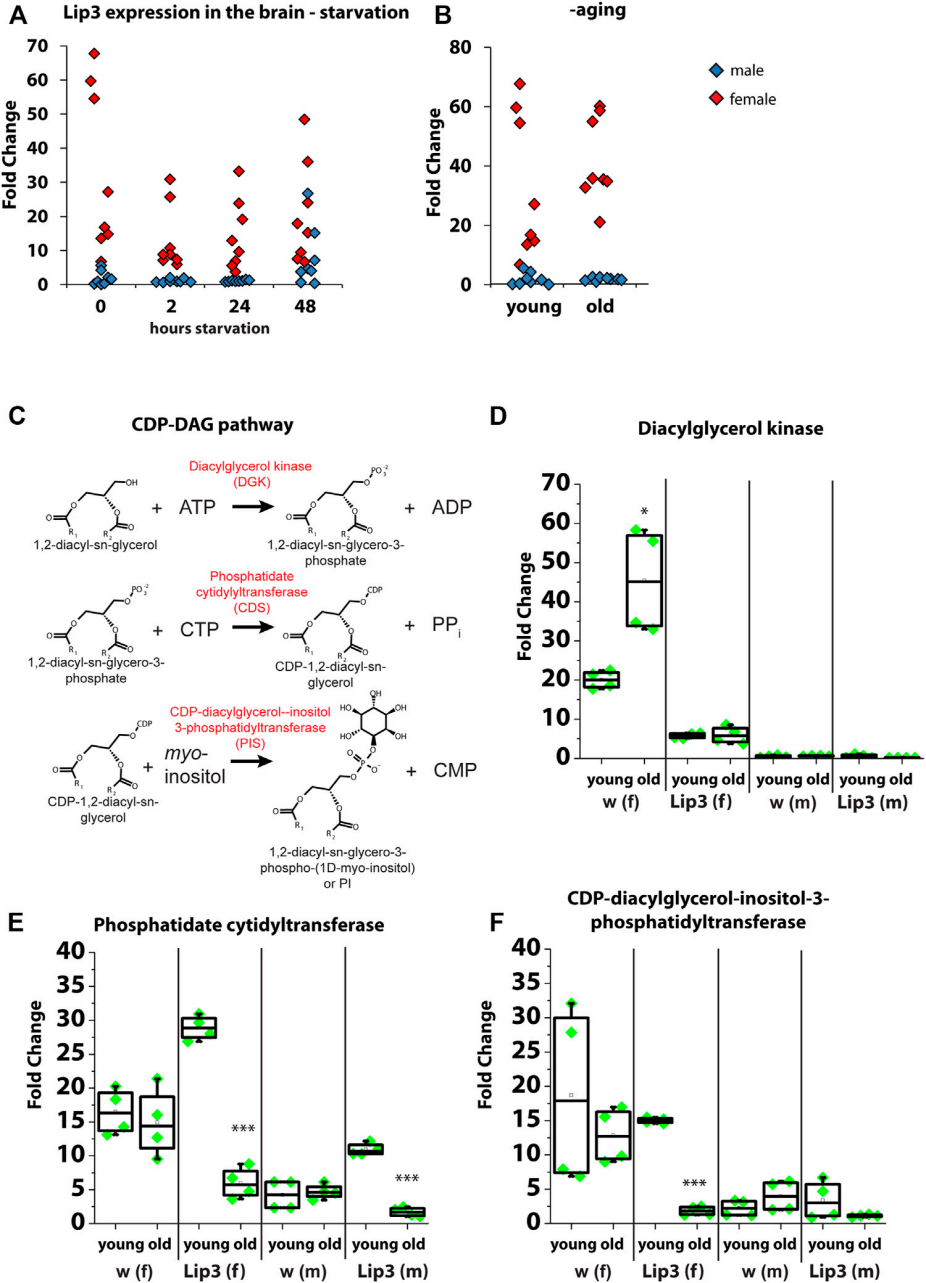
**(A)** Quantitative real-time PCR of Lip3 transcript in isolated adult brains from fed (0 h) and starved (2, 24, 48 h) male (blue squares) and female (red squares) normalized to rp49 and actin-5c housekeeping genes and relative to whole body from fed male and female flies, respectively. **(B)** Quantitative real-time PCR of Lip3 transcript in isolated adult brains from male (blue squares) and female (red squares) flies, normalized to rp49 and actin-5c housekeeping genes and relative to whole body from 5 day old male and female flies, respectively. Datasets for fed flies in A and young flies in B are identical. **(C)** Representation of the CDP-DAG pathway of PI synthesis. **(D)** Quantitative real-time PCR of DAG kinase (Dgk) transcript in isolated adult brains, normalized to rp49 and actin-5c housekeeping genes and whole body from 5 day old flies (male brains normalized to male whole flies and female brains normalized to female whole body). **(E)** Quantitative real-time PCR of phosphatidate cytidyltransferase (Cds) transcript in isolated adult brains, normalized to rp49 and actin-5c housekeeping genes and whole body from 5 day old flies (male brains normalized to male whole flies and female brains normalized to female whole body). **(F)** Quantitative real-time PCR of CDP-diacylglycerol-inositol-3-phosphatidyl-transferase (PIS) transcript in isolated adult brains, normalized to rp49 and actin-5c housekeeping genes and whole body from 5 day old flies (male brains normalized to male whole flies and female brains normalized to female whole body). Asterisks represent *p < 0.05, ***p < 0.001.

Our lipidomics analysis revealed an increase of PI in *Lip3* mutant larvae suggesting a function of the lipase in PI metabolism. PI gives rise to seven phosphoinositides by phosphorylation of its myo-inositol ring, which are important signaling molecules in the plasma membrane (Raghu et al., 2019). Since PI is enriched in the brain (Stillwell, 2016), we first tested *Lip3* expression in the brain of male and female flies under starvation and aging. We found that *Lip3* transcripts are enriched in the female brain relative to total body, but *Lip3* expression does not further increase upon starvation or aging (Figures 4A,B). In contrast, *Lip3* is only moderately enriched in the brain of male flies (compared to total body), but the gene expression rises slightly after 48 h of starvation. In brains of old flies, *Lip3* expression is not induced (Figures 4A,B). To investigate the regulation of PI in the brain, we analyzed the transcript levels of the enzymes of the CDP-DAG pathway of PI synthesis. DAG is converted to phosphatidic acid (PA) by Diacylglycerol kinase (Dkg). *Drosophila* Dgk regulates the secretion of insulin-like peptides from the insulin-producing cells in the brain (Trinh et al., 2019). PA is converted to CDP-DAG by CDP-diacylglycerol synthase (Cds, phosphatidate cytidyltransferase). This enzyme diverts PA from storage fat formation, a process required for cell growth during development (Liu et al., 2014). CDP-DAG is then combined with myo-inositol to yield PI, a process catalyzed by phosphatidylinositol synthase (PIS, CDP-diacylglycerol-inositol-3-phosphatidyl-transferase) (Figure 4C). Similar to *Lip3*, *Dgk* transcription is enriched in the female brain, but rises further in old flies. By contrast, brain enrichment is reduced and independent form age in *Lip3* mutant female brains. *Dgk* transcription is not enriched nor age-dependent in the brains of male flies. Both *Cds* and *PIS* transcription does not change significantly in the brain of female flies upon aging, but is reduced in the brain of old female Lip3 mutants. *Cds* transcription is also enriched in male brains, and drops significantly in old males. Together, our data suggest that the transcriptional regulation of PI synthesis is altered in aging *Lip3* mutants, mostly in aging Lip3 mutant female flies.

## Discussion

Dietary restriction prolongs lifespan by well-studied mechanisms, but when starvation progresses, an organism has to sacrifice vital molecules like structural lipids and proteins. Here we show that lipase 3 is highly upregulated under prolonged starvation and aging. Together with previous results, this suggests that high lipase 3 induction is a pathological mechanism that leads to lipotoxicity and mitochondrial damage (Bülow et al., 2018), while its suppression prolongs lifespan. This is in contrast to other lipases like Brummer, Lip1, Lip4 and Magro, that are transcriptionally regulated by Foxo (Grönke et al., 2005; Vihervaara and Puig, 2008; Karpac et al., 2013; Molaei et al., 2019): Magro is downregulated in old flies (Karpac et al., 2013), and downregulation of Brummer extends lifespan (Nazario-Yepiz et al., 2021). Foxo rapidly responds to low insulin signaling by nuclear shuttling, which induces the break-down of nutrient stores, gluconeogenesis and autophagy. Especially autophagy has been show to mediate lifespan extension upon caloric restriction (Madeo et al., 2015). We propose that signaling pathways with different effectors than Foxo mediate the response to prolonged starvation, and that regulation of these pathways is also affected in aging. Of note, Lip3 reduction shows a trend to prolong lifespan in male flies. Our study suggests that there are two phases of Lip3 transcriptional regulation: in response to short periods of nutrient deprivation, Lip3 is induced at moderate levels, which might be Foxo-dependent. As starvation continues, Lip3 induction increases to extreme levels, a process that is presumably Foxo-independent. Instead, two transcription regulators have been shown to regulate Lip3: Schlank and Hnf4 (Palanker et al., 2009; Sociale et al., 2018). Although we observed previously that ubiquitous or fat body-specific overexpression of Lip3 induces the formation of FFA (Bülow et al., 2018), our present study suggests that TAGs are not the substrate of Lip3-mediated lipid hydrolysis. Instead we propose that Lip3 acts as phospholipase that hydrolyzes primarily PI. PI is the precursor of the seven phosphoinositides, membrane phospholipids with important signaling function especially in the brain (Raghu et al., 2019). We suggest that Lip3 might be a regulator of PI levels in the brain, and that its expression has to be tightly regulated to prevent PI hydrolysis. Brain PI hydrolysis is linked to Alzheimer’s disease; we thus propose that high induction of Lip3 links prolonged starvation and aging, and that repression of Lip3 spares PI under both conditions. How exactly Lip3, or conditions that highly induce Lip3 transcription, affect phosphoinositide levels, remains to be determined. Our study suggests that high induction of Lip3, as observed in mutants with defective mitochondria or peroxisome biogenesis, has to be repressed under fed conditions as well as conditions of short starvation, where energy homeostasis by lipid mobilization is achieved by Foxo-regulated lipases. Further studies will reveal the regulatory networks that allow high Lip3 induction under extreme conditions and a possible impact of Lip3-mediated phospholipid hydrolysis in maintaining homeostasis under these conditions.

## Data Availability

The raw data supporting the conclusion of this article will be made available by the authors, without undue reservation.
