# Supplementary material for: Drosophila Lipase 3 Mediates the Metabolic Response to Starvation and Aging
Source: Front Aging. 2022 Feb 14;3:800153. doi: 10.3389/fragi.2022.800153 (PMC9261307; doi:10.3389/fragi.2022.800153)
Supplement: Supplementary file 1 [file DataSheet1.pdf]

Supplement

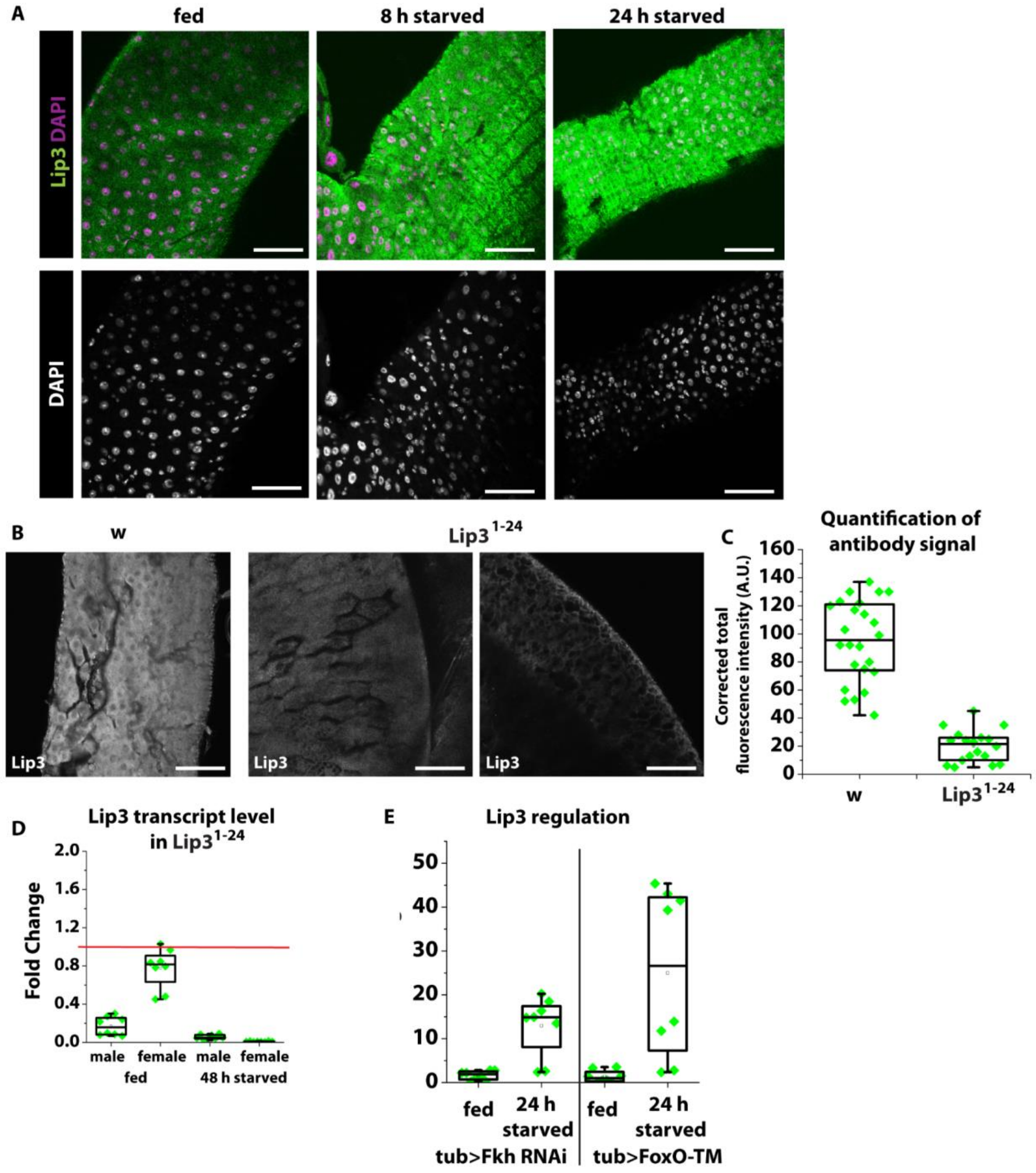

Supplemental Figure S1: A) Immunofluorescent staining of female midguts with anti-Lip3 in fed, 8 h starved, and 24 h starved flies. Upper panel shows merge of DAPI and Lip3 staining, lower panel shows DAPI. B) Immunofluorescent staining of female midguts with anti-Lip3 in fed wildtypes and Lip3 mutants. C) Quantification of fluorescent signal from B. D) Quantitative real-time PCR of starved male and female Lip3 mutants, normalized to their fed counterparts. F) Quantitative real-time PCR of Lip3 transcript of adult female flies, genotypes are +/+; tubulin Gal4/Fkh-RNAi and +/+; tubulin-Gal4/UAS-FoxoTM.
